# Tactile Preferences in Goats: Implications for Animal-Assisted Interventions

**DOI:** 10.3390/ani16050835

**Published:** 2026-03-07

**Authors:** Patrycja Magdalena Masier, Agnieszka Ziemiańska, Kamila Janicka, Wiktoria Janicka, Marta Wójcik, Iwona Rozempolska-Rucińska

**Affiliations:** 1 Institute of Biological Basis of Animal Production, University of Life Sciences in Lublin, Akademicka 13, 20-950 Lublin, Poland; patrycja.masier@up.edu.pl (P.M.M.); kamila.janicka@up.edu.pl (K.J.); wiktoria.janicka@up.edu.pl (W.J.); iwona.rucinska@up.edu.pl (I.R.-R.); 2 Oncology Lab, Department of Epizootiology and Clinic of Infectious Diseases, Faculty of Veterinary Medicine, University of Life Sciences in Lublin, Akademicka 13, 20-950 Lublin, Poland; marta.wojcik@up.edu.pl

**Keywords:** animal-assisted interventions, goat-assisted interventions, tactile contact, tactile preferences, human–animal relationship

## Abstract

Tactile contact is a key element of animal-assisted interventions (AAI), yet its effects on the animals involved remain poorly understood. Seven adult goats were included in the experiment, which examined three factors influencing tactile contact: the body region (head/neck, trunk, hindquarters; R1–R3), the ordinal position in the stroking sequence (first, second, third; O1–O3) and the location context (slightly enriched goat house and a highly enriched pasture; L1, L2). It was found that when stroking occurred first in the position of the sequence (O1), the duration of touch was significantly shorter (*p* < 0.0001). In addition, it was observed that when the session started with the trunk (R2), the total contact duration (T) was significantly longer (*p* < 0.039). No differences were found between the duration of contact in different locations (*p* = 0.179). These results may indicate that the initial phase was critical for the overall contact. Furthermore, the longer duration of contact, when initiated on the trunk, may indicate a relative preference for this body region. These findings suggest that human contact remains equally attractive to goats regardless of the enrichment of their location.

## Simple Summary

Touch is an important part of animal-assisted interventions, but little is known about how animals themselves respond to this type of contact. This study examined the effect of tactile contact on adult goats. It explored whether goats show preferences for specific body regions (head/neck, trunk, hindquarters), the ordinal position in the stroking sequence, and the environment in which the tactile interaction takes place (goat house vs. pasture). Goats were less receptive to the first touch in the sequence. In addition, goats showed longer contact durations when the stroking session started from the trunk. No preferences regarding the environment were detected. The initial touch may therefore be important in the context of the entire contact, and starting the touch from the trunk may be more readily accepted by the animal. Contact with humans remained attractive to goats even in an enriched environment.

## 1. Introduction

Touch is an essential element of communication in many animal species, facilitating bonding and influencing the regulation of emotions [1]. Research indicates that touch has a calming effect on humans and some other mammals [2]. Touch is associated with the release of oxytocin, and this response has been well documented in humans [3,4] and dogs [3,5]. It is believed that an animal’s acceptance of a human approach and the subsequent touch are clear indicators of a positive human–animal relationship (HAR) [6,7]. Significantly, touch is a key element of an animal-assisted intervention (AAI) [8].

The effects of animal-assisted interventions (AAI) on humans are well documented in the literature, with dogs and horses being the most frequently described species [9,10,11,12]. It has been demonstrated that the use of goats in this type of intervention can have positive psychosocial effects on humans [13,14]. In addition, it has been shown that even ordinary interactions with goats have a positive effect on the mental health of humans [15]. However, far fewer studies address the effect of AAIs on the animals themselves, and the results are not conclusive. It is the effect of AAIs on dogs that has been most frequently described [16,17]. To the authors’ knowledge, no studies describe the effect of AAIs on goats. There are, however, few publications describing the effect of interaction with goats on these animals, and, as a rule, their responses to touch are positive [18,19]. Ramont et al. [20] point out, however, that inappropriate touch can increase the number of agonistic behaviours towards humans. Physical contact is considered a crucial component of the effectiveness of AAI [8], and some authors suggest that stroking may be particularly important for achieving these effects [21]. McCullough et al. [22] noted that stroking a therapy dog was the most frequently chosen activity during an AAI session (92%). Beyond the context of AAI, stroking procedures in dairy goats have been associated with calmer reactions and greater comfort in the presence of humans [23]. Moreover, the quality of human handling has been shown to influence maternal behaviour [24], milk production [25], and weight gain [26].

The location of touch can play a significant role in shaping an animal’s response. Cows stroked on the ventral part of the neck showed more positive responses and behaved more calmly during veterinary procedures, compared to those stroked on the withers or the side of the chest [7,27,28]. It is worth noting that this region is often licked during social interactions among cattle [7,27]. In contrast, Lange et al. [29] showed no differences in responses between different stroking patterns in heifers. The animals responded positively to touching both the lower part of the neck and the entire neck and head region. As for horses, it has been demonstrated that touching within regions of the head, middle of the neck, and withers had a positive effect on both physiological and behavioural parameters as compared to touching within regions of other parts of the body [30,31]. The manner of touch also appears to be important, as horses preferred scratching the withers over patting on the neck [32]. Importantly, the withers are the preferred region to be touched during mutual grooming in horses [33]. It has also been demonstrated that gentle grooming of the body regions preferred by horses increased their willingness to interact with humans compared to those subjected to the standard procedure [34]. Pigs exhibited positive neurophysiological and behavioural responses when stroked on the abdomen, although other regions of the body were not compared [35]. Dogs exhibited more anxiety- and stress-related reactions when they were stroked around the head or shoulders, or when a human held their paw [36,37]. In contrast, McGreevy et al. [38] obtained opposite results, as stress indicators were significantly lower regardless of the body region. Dogs also preferred stroking to scratching or patting, as indicated by biochemical parameters [39]. Cats responded positively to being touched within the temporal region [40], whereas the strongest negative behavioural responses were caused by touching the tail [40,41] as compared to other regions. The implementation of the cat stroking protocol, developed by Haywood et al. [42], resulted in an increase in the number of positive behavioural responses. It is worth noting, however, that the response to touch in a particular body region is species-specific and cannot be generalized across species.

Small ruminants represent species that have been poorly researched in terms of their preferences for specific regions of the body. Stroking sheep within the neck, withers or forehead regions, or brushing various regions of the body (ventral part of the neck, sides of the chest, and withers), produced positive behavioural and physiological responses [43,44]. Stroking goats on the back and brushing their back and trunk had a positive effect on their behavioural and physiological responses [18,19]. It should be noted, however, that none of the studies on small ruminants, cited above, compared the individual body regions with each other.

There is limited literature addressing the effect of position within a touching sequence on animals’ responses. Kuhne et al. [36] noted that test sequences significantly affected dogs’ behavioural responses. In contrast, Ellis et al. [41] observed no significant differences in the order of stroking individual body regions in cats. The variability of the results indicates that the ordinal position within the sequence may be important for interpreting the actual preferences of animals.

Individual animals differ in the way they respond to stimuli, and these responses are repeatable over time and in different situations [45,46,47]. This idea is described by temperament [46,48] which, in a model proposed by Réale et al. [46], includes five categories: shyness–boldness, exploration-avoidance, activity, sociability and aggressiveness. The dimension of shyness–boldness is particularly important as it concerns responses to a human, which is the subject of analysis in this paper. It should be noted, however, that this dimension is broader and also concerns responses to risky situations, excluding responses to new situations [46,49,50]. It has been demonstrated that goats with higher boldness levels exhibited more aggressive behaviours when fed and learned certain tasks less effectively than shy animals [51,52]. It has also been indicated that shy goats had significantly higher stress markers in situations of discomfort and exhibited a higher tendency to milk secretion disorders [25,53]. Leite et al. [54] observed individual preferences in goats being stroked but did not report detailed quantitative data. It was particularly interesting to find out that goats relied more frequently on personal information than social information in conflicting situations [55]. This may contribute to greater behavioural diversity and, possibly, to greater expression of temperamental traits, but further research is needed in this area.

Many researchers point out that a richer environment draws positive responses in animals. Sheep kept on pasture showed significantly lower physiological and behavioural stress indicators [56], and significantly better results in cognitive bias tests [57], as compared to measurements taken in the sheepfold. Similarly, rabbits staying in a rich environment performed better in behavioural tests [58]. It is worth noting, however, that the impact of the environment on animal behaviour is not always unequivocally positive. It has been observed that pigs in a rich environment felt more anxiety when encountering a novelty item or situation and were less involved in social contacts [59]. This behaviour has also been noted in parrots [60]. In addition, Górecki et al. [61] demonstrated that goats exhibited affiliative behaviour significantly more frequently in the goat house than on the pasture, where the number of interactions was significantly lower. It is, therefore, possible that an overly attractive location reduces the number of pro-social behaviours.

The authors formulated a hypothesis that a goat’s willingness to come into contact with humans is determined by region and position of touch and the conditions under which the interaction takes place. They assumed that the duration of contact may vary depending on the body region being stroked and the ordinal position within the sequence. They also assumed that the environment in which the animal is staying and its enrichment may affect the animal’s willingness to interact with a human. This study aimed to determine the general predispositions and individual preferences of goats used in animal-assisted interventions (AAI).

## 2. Materials and Methods

### 2.1. Study Group

All experimental procedures were approved by the Local Ethics Committee for Animal Experimentation of Lublin, Poland (Approval No 8/2025).

The study involved seven adult goats (*n* = 7) used in animal-assisted interventions. The animals were aged between 1 and 6 years. The goats had no specific breed origin. None of the goats were used for production purposes, and their care allowed them to explore freely and have daily contact with humans. The animals were kept under a pen-and-pasture system. During the non-grazing season, the goats were housed in a goat house. The goat house had an area of 60 m^2^ and consisted of two rooms bedded with wheat straw. It also included an outdoor enclosure with an area of 100 m^2^, which connected the two rooms. The goat house was equipped with automatic drinkers, hay racks, wooden climbing platforms, and scratching brushes. During the grazing season, the goats were kept on pasture. The grass pasture covered an area of 1ha and was enclosed with a 1.5 m high metal mesh fence. The pasture included wooden shelters (each with an area of 20 m^2^) bedded with wheat straw and equipped with hay racks. There was also a strip of fruit trees approximately 10 m wide, which extended along the entire length of the pasture. In addition, the pasture was equipped with watering sites, scratching brushes and a playground consisting of a small house and climbing structures. Two caretakers alternated in caring for the animals. They were responsible for the daily care of the goats, including the preparation of concentrated feed, hay delivery, feeding, providing fresh water and bedding, and manure removal. The goats were fed once daily with a mixture of root vegetables (carrots and beetroots), dried herbs, and concentrated feed for small ruminants. Meadow hay was provided in quantities sufficient for one day. All goats were regularly dewormed, vaccinated, and had their hooves trimmed. All the goats were subject to regular veterinary inspections.

### 2.2. The Course of the Experiment

The gentle stroking procedure involved a slow movement of open palms over the animal’s body, with the fingers moving simultaneously in a gentle manner (without applying pressure). The movement was carried out along the coat. As for long-haired goats, the movement of the palm was adjusted to avoid pulling the hair, likely to cause discomfort. All tests were conducted by a single experimenter who was familiar to the animals. The procedure was initiated with a standard approach, and then the duration of contact was recorded. During all tests, the entire herd remained in the location of their choice. The tests were conducted every other day at different times, depending on organizational conditions. Time was measured using a digital stopwatch.

#### 2.2.1. Assessment of the Effect of the Body Region and the Ordinal Position

**Figure 1:**
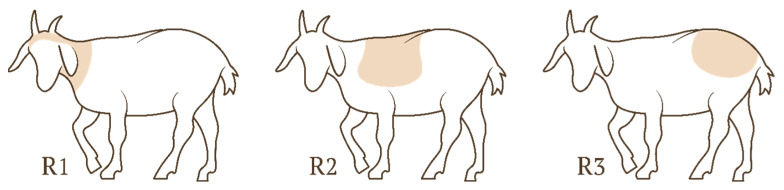
Diagram for the location of the three stroking regions. Legend: body regions 1 (R1), 2 (R2), and 3 (R3).

This stage of the experiment was conducted in the goat house during the winter. Throughout that period, all the animals were kept permanently in the goat house. During that stage, each goat was stroked on three distinguished body regions (R1–R3) (Figure 1) (Table 1). Each goat was stroked only once on a particular day, in a single session involving all three regions (R1, R2, R3). The body regions were stroked sequentially, and each body region could occur in any of the three ordinal positions within the sequence: the first (O1), second (O2), and third (O3). Accordingly, nine body region × position combinations were defined. The sequence of body regions was predetermined and remained the same for all animals on a particular day. Each region was stroked for a maximum of 90 s, which implies that the entire session could last up to 270 s (total stroking duration, T). During a session, a goat could walk away from the experimenter three times. Once a goat walked away from the experimenter, the time measurement for all parameters (R, O) was stopped. The experimenter then approached the animal again and began a new approach from the next body region in the predetermined sequence. The sessions were completed after the animal walked away for the third time, or after the last body region had been stroked. A total of 24 sessions were conducted for each individual.

#### 2.2.2. Assessment of the Effect of Location

The second stage of the study was conducted at two different locations: a slightly enriched one (goat house, L1) and a highly enriched one (pasture, L2). First, a test was carried out in the goat house, which was the animals’ permanent place of stay during the winter. The test was conducted in March. After the grazing season started in April, the test was then conducted on the pasture. Before the tests began, the goats acclimatized for two weeks on the pasture. The stroking procedure was all-inclusive, as it involved all pre-defined body regions, and lasted for up to 270 s. The session was terminated when the animal walked away or when the time limit was reached. Each goat was stroked only once on a particular day. A total of 20 tests were conducted on each individual: 10 at location L1 and 10 at location L2.

### 2.3. Statistical Analysis

The analyses were performed using the GLIMMIX procedure in SAS 9.4 (SAS Institute, Cary, NC, USA), applying a generalized linear mixed model. The model included the following fixed effects: body region, ordinal position in the sequence, the body region and ordinal position interaction, individual, the individual and body region interaction and the order of the test day as a regression term. *p*-values for pairwise comparisons of LSMeans were adjusted using Tukey’s method. The same modelling approach was used to evaluate the effect of location. Compact letter display was determined in the R 4.5.1 programme (The R Foundation for Statistical Computing, Vienna, Austria) based on the significance testing results (α = 0.05).

## 3. Results

### 3.1. Differences Between Body Regions and the Ordinal Positions

**Figure 2:**
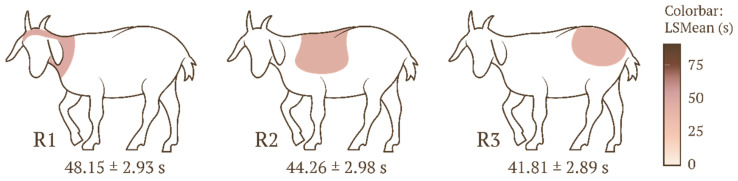
Mean stroking time (LSMeans) and standard error (SE) depending on the body region; the degree of shading corresponds to the mean stroking time. Legend: body regions 1 (R1), 2 (R2), and 3 (R3).

**Figure 3:**
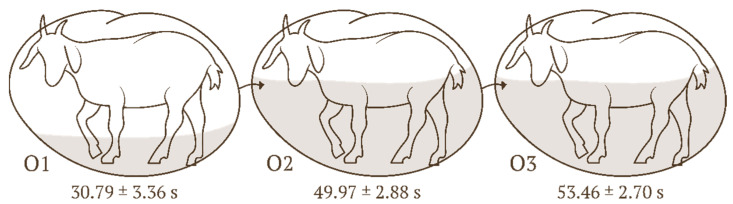
Mean stroking time (LSMeans) and standard error (SE) depending on the ordinal position. Legend: ordinal positions in the sequence 1 (O1), 2 (O2), and 3 (O3).

All the values are presented as least squares means (LSMeans) and standard error (SE), unless otherwise indicated. No significant effect of the body regions (R1, R2, R3) on the duration of animal stroking was observed (F(2, 489) = 1.500, *p* = 0.225, ηp^2^ = 0.006) (Figure 2). A significant effect of the ordinal position within the sequence (O1, O2, O3) on the stroking duration was noted (F(2, 489) = 19.750, *p* < 0.0001, ηp^2^ = 0.075). The stroking duration for O1 (30.79 ± 3.36 s) was significantly shorter (*p* < 0.0001) than the duration for both O2 (49.97 ± 2.88 s) and O3 (53.46 ± 2.70 s) (Figure 3).

**Figure 4:**
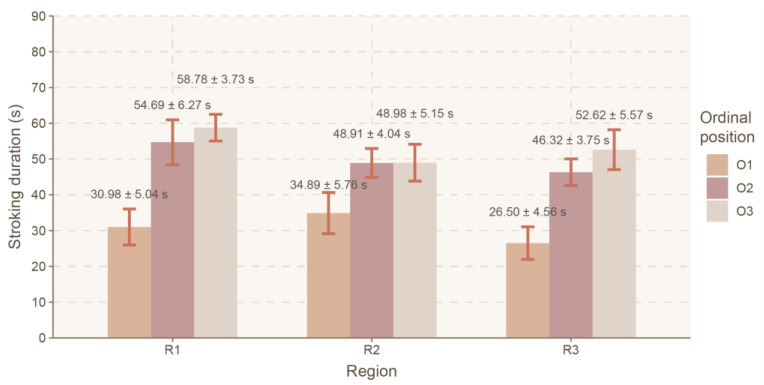
Mean stroking time (LSMeans) and standard error (SE) depending on the body region and the ordinal position. Legend: body regions 1 (R1), 2 (R2) and 3 (R3); ordinal positions in the sequence 1 (O1), 2 (O2) and 3 (O3).

No significant effect of interaction between the body region and the ordinal position was noted (F(4, 489) = 0.380, *p* = 0.824, ηp^2^ = 0.003) (Figure 4).

**Figure 5:**
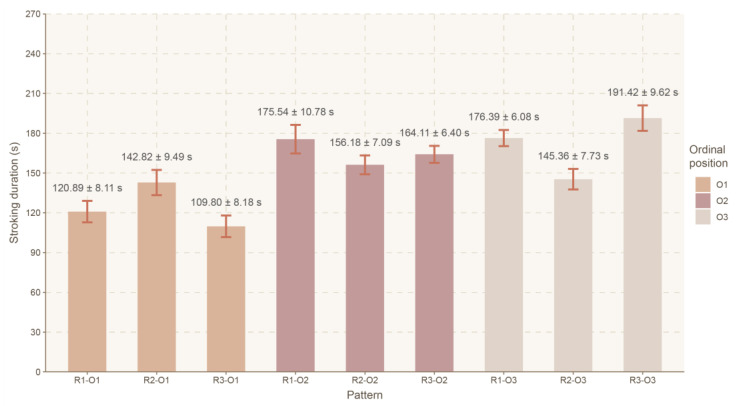
Mean total stroking time (LSMeans) and standard error (SE) depending on the stroking pattern. Legend: each pattern is a combination of one of the three body regions (R1, R2 and R3) and one of the three ordinal positions within the sequence (O1, O2 and O3).

The stroking pattern (a combination of R and O) had a significant effect on its total duration (T) (F(8, 484) = 10.550, *p* < 0.0001, ηp^2^ = 0.148). Analysis of ordinal position within body regions showed that sessions starting from the trunk (R2-O1) (142.82 ± 9.49 s) had a significantly higher T value (*p* < 0.039) than those starting from the head/neck (R1-O1) (120.89 ± 8.11 s) or from the hindquarters (R3-O1) (109.80 ± 8.18 s). In turn, sessions ending on the trunk (R2-O3) (145.36 ± 7.73 s) showed a significantly lower T value (*p* < 0.002) than those ending with the head/neck (R1-O3) (176.39 ± 6.08 s) or with the hindquarters (R3-O3) (191.42 ± 9.62 s). Analysis of the body regions in a particular position showed that when the head/neck was stroked as the first region (R1-O1) (120.89 ± 8.11 s), the T was significantly shorter (*p* < 0.0001), compared to the configuration when the head/neck was stroked as the second (R1-O2) (175.54 ± 10.78 s) or the third region (R1-O3) (176.39 ± 6.08 s). Where the hindquarters were stroked as the first region (R3-O1) (109.80 ± 8.18 s), T was significantly shorter (*p* < 0.0001) than in the cases where they were stroked as the second (R3-O2) (164.11 ± 6.40 s) or the third region (R3-O3) (191.42 ± 9.62 s) (Figure 5).

**Figure 6:**
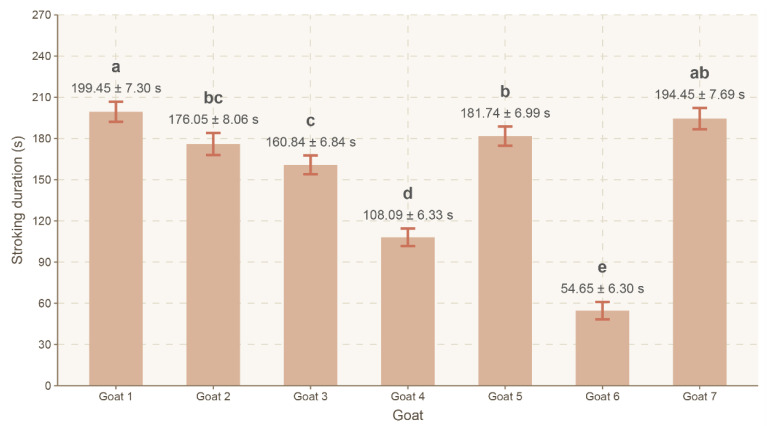
Mean total stroking time (LSMeans) and standard error (SE) depending on the individual (goats 1–7).

A significant effect of an individual on the total stroking duration (T) was noted (F(6, 484) = 56.330, *p* < 0.0001, ηp^2^ = 0.411). Goat 1 exhibited the highest T value (199.45 ± 7.30 s), which was significantly higher than that in goats 2–6 (*p* < 0.042). Goat 7 (194.45 ± 7.69 s) showed a significantly higher T value than goats 3, 4 and 6 (*p* < 0.0003). Goat 5 (181.74 ± 6.99 s) exhibited a significantly higher T value than goats 3, 4 and 6 (*p* < 0.016). Goat 2 (176.05 ± 8.06 s) showed a significantly higher T value than goats 4 and 6 (*p* < 0.0001). Goat 3 (160.84 ± 6.84 s) exhibited a significantly higher T value than goats 4 and 6 (*p* < 0.0001). Goat 4 (108.09 ± 6.33 s) showed a significantly higher T value than goat 6 (*p* < 0.0001). Goat 6 exhibited the lowest T value (54.65 ± 6.30 s). The compact letter display applied indicates the existence of five distinct statistical groups (a–e) (Figure 6).

**Figure 7:**
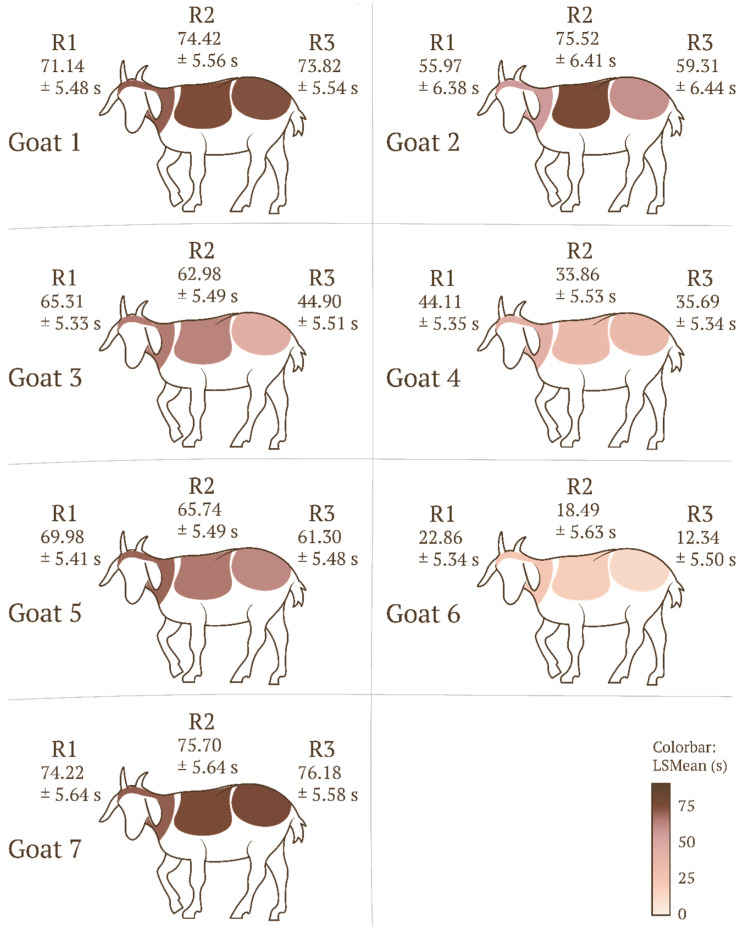
Mean total stroking time (LSMeans) and standard error (SE) depending on the body region in individual animals; the degree of shading corresponds to the mean stroking time. Legend: body regions 1 (R1), 2 (R2), and 3 (R3).

A significant effect of interaction between the body region and an individual was observed (F(20, 477) = 12.270, *p* < 0.0001, ηp^2^ = 0.340). For goat 2, the duration of stroking the trunk (R2) (75.53 ± 6.41 s) was significantly longer (*p* = 0.027) than that for stroking the head/neck (R1) (55.97 ± 6.38 s). For goat 3, the duration of stroking the hindquarters (R3) (44.90 ± 5.51 s) was significantly shorter (*p* = 0.017) than that for stroking the head/neck (R1) (65.31 ± 5.33 s) and that for stroking the trunk (R2) (62.98 ± 5.49 s). For the remaining goats, no significant differences were noted for this parameter (*p* > 0.05) (Figure 7).

### 3.2. Differences Between Locations

**Figure 8:**
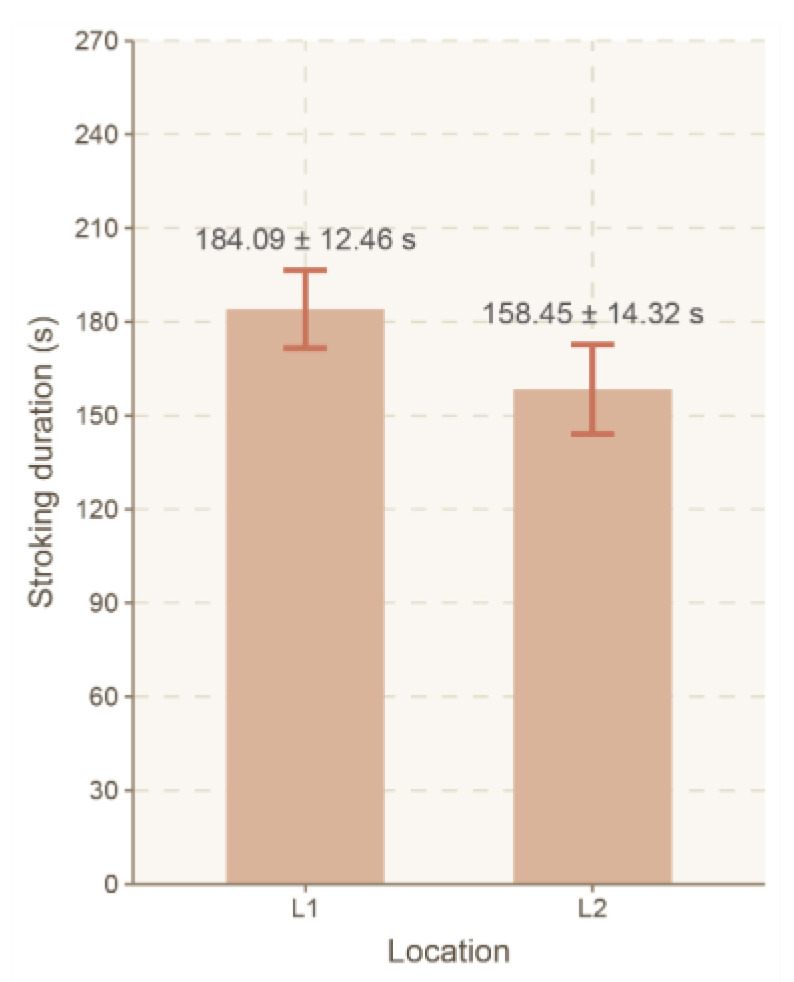
Mean total stroking duration (LSMeans) and standard error (SE) depending on location enrichment. Legend: location 1 (L1)—goat house (L1); location 2 (L2)—pasture.

No significant effect of location (L1, L2) on the duration of animal stroking was observed (F(1, 121) = 1.820, *p* = 0.179, ηp^2^ = 0.015) (Figure 8).

## 4. Discussion

### 4.1. Effect of the Body Region on Touch Duration

The present study assumed that the body region could affect the goat’s response when stroked, but the results did not confirm this hypothesis. The duration of animal stroking did not differ significantly between the distinguished regions, namely, the head/neck, the trunk, and the hindquarters (*p* = 0.225). Interesting results were obtained when analyzing the impact of individual stroking patterns on the total duration of the session. Sessions starting from the trunk (R2) were significantly longer than those starting from other body regions (*p* < 0.039). The authors believe that the first ordinal position (O1) could have been of critical importance for the study. It was probably mainly at that stage that the animals decided whether they wanted to continue interacting. During subsequent stages (ordinal positions O2 and O3), a sense of predictability may have been more important to the goats, potentially reducing the significance of a specific body region. This means that, actually, the trunk could have been the most preferred region, but the effect would only be noticeable initially. An interesting direction for research into the willingness to be touched is the choice of body region touched during allogrooming, which has been positively received by other species [7,27,33]. The most common affiliative behaviours between goats include lying next to each other (trunk-trunk) and touching each other’s muzzles [61,63], which would only be partially consistent if the trunk was preferred. Some authors note, however, that goats exhibit a lower tendency towards intraspecific affiliative behaviour as compared to other species [64]. Furthermore, due to the different nature of interspecific interactions, not all forms of touch accepted in interactions between goats are necessarily equally well tolerated in interactions with humans. The observed effect may have practical implications for AAI. As noted by Kaminski et al. [21], stroking may be crucial to the effectiveness of AAI. Identifying body regions preferred by animals may significantly influence the course of the entire session, the animals’ willingness to remain in contact with humans, and thus their subsequent well-being. Associating the circumstances of the session with pleasant experiences in goats may facilitate interactions with patients in the future. We therefore believe that this result may be important in the context of using goats in AAI. However, further research is needed to determine whether there is a common pattern of tactile preferences in goats.

### 4.2. Effect of the Ordinal Position on Touch Duration

The authors formulated a hypothesis that the ordinal position within the sequence could affect the duration of stroking, which was confirmed by the results. A decrease in the duration of interaction across subsequent stages (the ordinal positions) could be expected due to a gradual loss of interest. The effect, however, was the opposite, as it was demonstrated that the touch that occurred first in the sequence (O1) lasted the shortest (*p* < 0.0001) compared to the other ordinal positions. The authors believe it was at that stage that the goats most often took the opportunity to walk away from the experimenter, thus deciding whether to continue or discontinue their interaction with a human. It appears that early termination of contact may have indicated the animal’s uncertainty about the experimenter’s intentions. The experimenter themselves could be either an aversive stimulus or both an appetitive and an aversive one, thus creating an approach–avoidance conflict in the animal. This conflict involves assessing the costs and benefits in a situation where opposing incentive systems operate simultaneously [65,66]. On the other hand, however, the interaction may simply have been considered unattractive at that particular moment. The subsequent stages of the session (ordinal positions O2 and O3) could be perceived as more predictable and also reflect the responses of goats that were more willing to interact. Subsequently, goats remaining in contact could undergo habituation [67] within the session, which, in turn, likely resulted in even longer interaction duration. Bejder et al. [68] report, however, that the term habituation is often overused, and, in many cases, a moderate response of an animal is only associated with an increase in its individual tolerance to the stimulus [69].

### 4.3. Effect of Individual Variability on the Duration of Touching

The present study assumed that individual differences between the goats could affect the duration of an interaction, which was confirmed by the results. The compact letter display enabled the identification of five statistical groups (a–e) based on the total stroking duration (T). Although this study did not use a standardized test to assess temperament, the outlined groups may indicate the direction of the emergence of the shyness–boldness dimension (from e to a). The authors believe that only further research will show whether these trends are consistent over time and across different contexts.

An interesting result was the observation of differences in contact duration depending on the body region in goats 2 and 3. Goat 2 remained in contact longer when stroked on the trunk (R2) as compared to the head/neck (*p* < 0.027). However, in goat 3, the contact lasted longer when the head/neck (R1) and the trunk (R2) were stroked as compared to stroking the hindquarters (R3) (*p* < 0.017). The authors believe that the goats had their preferences as regards the body region to be stroked. It is worth noting that an animal’s individual traits can significantly affect the human–animal relationship (HAR) [70]. A successful relationship involves both a mutual desire for contact and specific types of communication and socio-cognitive competence in the animal [6]. Research has shown that goats differed in their responses to humans, depending on their location on the shyness–boldness axis spectrum. A significant correlation has been found between high levels of shyness and a negative response to a human [25,71]. It should be noted that HAR is a dynamic and mutual process modulated by individual and contextual factors [6,72].

### 4.4. Effect of Location on the Duration of Touching

The present study assumed that location enrichment could affect the duration of an interaction. This assumption, however, was not confirmed by the results. The duration of stroking did not differ significantly between the goat house and the pasture (*p* = 0.179). This result is interesting, considering that the pasture (L2) was a much richer environment compared to the goat house (L1); therefore, the authors expected a trend towards a decrease in the duration of interaction. The authors believe that interaction with humans in the form of stroking could have been such an attractive stimulus that it surpassed all the other stimuli found in the animals’ environment. The pasture was much larger than the goat house and also offered an orchard and even a playground for the goats. Nevertheless, despite numerous stimuli, the goats’ motivation to interact with humans remained constant. It therefore appears that goats may be a species highly oriented towards contact with humans and thus predisposed to AAI.

### 4.5. Study Limitations

In the present study the tests conducted in the goat house took place earlier in the experimental timeline. Consequently, the goats may have become accustomed to the stroking procedure, which could have reduced the potential differences between the locations. Moreover, it has been shown that goats exhibit significant differences in behaviour depending on the season [73]. On the other hand, the study design was aligned with a pen-and-pasture system. Altering this sequence would have required changes to routine husbandry practices and could have been stressful for the goats. It is also worth noting that the experimenter was a person familiar to the animals. In most AAI settings, however, participants are unfamiliar to the animals; therefore, the findings should be interpreted with caution. On the other hand, Menna et al. [74] emphasize that the effectiveness of AAI depends on the relationship between the handler, the animal, the patients, and any other healthcare professionals involved in the process. Nevertheless, these findings cannot be directly generalized to patients. Walking away from a human appears to be a useful indicator of a lack of willingness to interact. Tests based on avoidance responses are a basic tool for assessing human-goat interaction [75]. However, walking away from a human may also have been related to other motivations, such as the approach of another goat or distraction by external factors. It is therefore possible that, in some cases, the interaction was interrupted despite the goat’s willingness to continue.

## 5. Conclusions

The authors of the present study demonstrated that the ordinal position in the stroking sequence significantly affected the duration of interaction, with the shortest contact time noted for the first position in the sequence. It appears that the initial phase was decisive for the entire interaction. It was also noted that when stroking began with the trunk, the entire session lasted significantly longer (compared to the head/neck and the hindquarters), which may indicate a relative preference for this region. The authors also demonstrated that the location had no significant effect on the duration of contact, suggesting that interaction with a human remained attractive regardless of environmental conditions. These conclusions suggest the potential suitability of goats for use in AAIs, although this needs to be confirmed through further research.

## Figures and Tables

**Table 1:** Anatomical descriptions of individual stroking regions in accordance with Veterinaria Nomina Anatomica [62].

Designation	Operational Name	Anatomical Description
R1	head/neck	The region included the regions of the head and neck (regiones capitis et regiones colli): the frontal region (regio frontalis), the parietal region (regio parietalis), the temporal region (regio temporalis), the horn region (regio cornualis), the occipital region (regio occipitalis), the dorsal collar region (regio colli dorsalis), the lateral collar region (regio colli lateralis), and the ventral collar region (regio colli ventralis). The remaining regions of the head and neck, including the facial region (regiones faciei) and the auricular region (regio auricularis), were excluded. The horns (cornua) were excluded.
R2	trunk	The region included the pectoral and dorsal regions (regiones pectoris et dorsi): the scapular region (regio scapularis), the costal region (regio costalis) and the thoracic vertebral region (regio vertebralis thoracis). The remaining pectoral and dorsal regions, including the presternal region (regio presternalis), the sternal region (regio sternalis) and the cardiac region (regio cardiaca), were excluded.
R3	hindquarters	The region included the dorsal and pelvic regions (regiones dorsi et pelvis): the lumbar region (regio lumbalis), the sacral region (regio sacralis), the gluteal region (regio glutea) and the clunial region (regio clunis). Additionally, it included areas from the lumbar region in the caudal direction to the hindquarters region, and from the midline of the back (linea mediana dorsalis) in the ventral direction to the course of the costal arch (arcus costalis). The remaining dorsal and pelvic regions, including the caudal region (regio caudalis) and the perineal region (regio perinealis), were excluded.

## Data Availability

The original contributions presented in this study are included in the article. Further inquiries can be directed to the corresponding author.

## Abbreviations

The following abbreviations are used in this manuscript:

HAR	Human–Animal Relationship
AAI	Animal-Assisted Intervention
R1–R3	Body regions: 1 (head/neck, R1), 2 (trunk, R2), and 3 (hindquarters, R3)
O1–O3	Ordinal position in the stroking sequence: 1 (first, O1), 2 (second, O2), and 3 (third, O3)
L1–L2	Location: 1 (goat house, L1), 2 (pasture, L2)
T	Total stroking duration
